# *Sinocoelotes* gen. n., a new genus of the subfamily Coelotinae (Araneae, Agelenidae) from Southeast Asia

**DOI:** 10.3897/zookeys.614.8663

**Published:** 2016-09-01

**Authors:** Lu Chen, Zhe Zhao, Shuqiang Li

**Affiliations:** 1 Institute of Zoology, Chinese Academy of Sciences, Beijing 100101, China; 2 Southeast Asia Biodiversity Research Institute, Chinese Academy of Sciences, Menglun, Mengla, Yunnan 666303, China

**Keywords:** Taxonomy, description, diagnosis, morphology, new combination, China

## Abstract

A new genus of the spider subfamily Coelotinae, *Sinocoelotes* **gen. n.**, with nine new species, is described from Yunnan and Sichuan Provinces in southern China. The new species are: *Sinocoelotes cangshanensis* **sp. n.** (♀), *Sinocoelotes hehuaensis* **sp. n.** (♂♀), *Sinocoelotes luoshuiensis* **sp. n.** (♀), *Sinocoelotes mangbangensis* **sp. n.** (♀) from Yunnan; *Sinocoelotes kangdingensis* **sp. n.** (♀), *Sinocoelotes ludingensis* **sp. n.** (♂♀), *Sinocoelotes mahuanggouensis* **sp. n.** (♀), *Sinocoelotes muliensis* **sp. n.** (♀), and *Sinocoelotes yanyuanensis* **sp. n.** (♂) from Sichuan. In addition, six *Coelotes* species are transferred to the new genus: *Sinocoelotes acicularis* (Wang, Griswold & Ubick, 2009), **comb. n.** (♂♀), *Sinocoelotes forficatus* (Liu & Li, 2010), **comb. n.** (♂♀), *Sinocoelotes guangxian* (Zhang, Yang, Zhu & Song, 2003), **comb. n.** (♂♀), *Sinocoelotes pseudoterrestris* (Schenkel, 1963), **comb. n.** (♂♀), *Sinocoelotes pseudoyunnanensis* (Wang, Griswold & Ubick, 2009), **comb. n.** (♂♀) and *Sinocoelotes thailandensis* (Dankittipakul & Wang, 2003), **comb. n.** (♂♀). DNA barcodes of all the species were documented for future use.

## Introduction

Coelotine spiders are common in the Northern Hemisphere. So far, a total of 662 valid species belonging to 25 genera (Wang 2002, Chen et al. 2015a, Chen et al. 2015b, Jiang and Chen 2015, Chen et al. 2016, Zhao and Li 2016) are known in the Holarctic and Southeast Asia. Twenty-two genera of Coelotinae are known from Asia. Among them, 18 genera including 294 species are reported from China (the most species-rich region) (Li and Lin 2016). Coelotinae from China are partly revised (Ovtchinnikov 1999, Wang 2002, Wang 2003, Chen et al. 2015a, Chen et al. 2015b, Chen et al. 2016); however, some genera and species remain poorly studied. The most species-rich genus remaining *Coelotes* Blackwall, 1841, seems to be polyphyletic.

The genus *Coelotes* was described by Blackwall (1841) for *Clubiona saxatilis* Blackwall, 1833, which was later synonymized with *Drassus atropos* Walckenaer, 1830. In the recent years, the genus was mainly revised by Ovtchinnikov (1999, 2000) and Wang (2002). They described 12 new genera and subgenera: *Asiacoelotes* Wang, 2002 (now considered to be a junior synonym of *Iwogumoa*), *Bifidocoelotes* Wang, 2002, *Brignoliolus* (subgenus) Ovtchinnikov, 1999, *Eurocoelotes* Wang, 2002 (now considered to be a junior synonyms of *Inermocoelotes*), *Femoracoelotes* Wang, 2002, *Himalcoelotes* Wang, 2002, *Inermocoelotes* Ovtchinnikov, 1999, *Leptocoelotes* Wang, 2002, *Platocoelotes* Wang, 2002, *Spiricoelotes* Wang, 2002, *Tegecoelotes* Ovtchinnikov, 1999, and *Urocoras* Ovtchinnikov, 1999. Currently, 184 species are included in *Coelotes* (World Spider Catalog 2016), but the genus still remains polymorphic; for example, the epigynal teeth are present in *Coelotes atropos* (Walckenaer, 1830), but absent in *Coelotes ningmingensis* Peng, Yan, Liu & Kim 1998; the epigynal hoods are present in *Coelotes septus* Wang, Yin, Peng & Xie 1990, but absent in *Coelotes terrestris* (Wider, 1834). In general, *Coelotes* is an extremely heterogeneous genus. To improve the systematic composition of *Coelotes*, further work needs to be done.

In this paper, a new genus of coelotine spiders, *Sinocoelotes* gen. n. and nine new species from China are described, and six new combinations are suggested.

## Material and methods

Specimens were examined with a LEICA M205C stereomicroscope. Images were captured with an Olympus C7070 wide zoom digital camera (7.1 megapixels) mounted on an Olympus SZX12 dissecting microscope. Epigynes and male palps were examined after dissection from the spiders’ bodies. Epigyne was cleared by boiling it in 10% KOH solution before take photos of the vulva.

All measurements were obtained using a LEICA M205C stereomicroscope and are given in millimeters. Leg measurements are shown as: Total length (femur, patella + tibia, metatarsus, tarsus). Only structures (palp and legs) of the left body side were described and measured. The terminology used in the text and the figure legends follows Wang (2002). Abbreviations used in this paper and in the figure legends: A = epigynal atrium; ALE = anterior lateral eye; AME = anterior median eye; AME-ALE = distance between AME and ALE; AME-AME = distance between AME and AME; ALE-PLE = distance between ALE and PLE; C = conductor; CD = copulatory duct; CDA = dorsal conductor apophysis; CF = cymbial furrow; E = embolus; EB = embolic base; ET = epigynal teeth; FD = fertilization duct; LTA = retrolateral tibial apophysis; MA = median apophysis; PA = patellar apophysis; PLE = posterior lateral eye; PME = posterior median eye; PME-PLE = distance between PME and PLE; PME-PME = distance between PME and PME; RTA = retro-ventral tibial apophysis; S = spermatheca; SA = anterior part of spermatheca; SH = spermathecal head; SP = posterior part of spermatheca; ST = subtegulum; T = tegulum.

Abbreviations used for museums and other institutions: CAS = California Academy of Sciences, San Francisco, USA; HNU = Hunan Normal University, Changsha, China; IZCAS = Institute of Zoology, Chinese Academy of Sciences, Beijing, China; MHBU = Museum of Hebei University, Baoding, China; MHNG = Muséum d’Histoire Naturelle, Geneva, Switzerland; MNHP = Muséum National d’Histoire Naturelle, Paris, France.

DNA barcodes were obtained for future use. A partial fragment of the mitochondrial cytochrome oxidase subunit I(COI) gene was amplified and sequenced for 15 species (all nine new species and six species, for which we introduced new combinations) using Primers: LCO1490-oono (5’-CWACAAAYCATARRGATATTGG-3’) (Folmer et al. 1994) and HCO2198-zz (5’-TAAACTTCCAGGTGACCAAAAAATCA-3’) (Zhao and Li 2016). For additional information on extraction, amplification and sequencing procedures, see Zhao et al. (2013). All sequences were analyzed using BLAST and are deposited in GenBank. The accession numbers are provided in Table 1.

**Table 1.** Voucher specimen information.

Species	GenBank accession number	Sequence length	Collection localities
*Sinocoelotes acicularis* (Wang et al., 2009)	KX555516	630bp	Lushui County, Yunnan Province, China
*Sinocoelotes cangshanensis* sp. n.	KX555514	630bp	Hehua Village, Xiaguan Town, Yunnan Province, China
*Sinocoelotes forficatus* (Liu & Li, 2010)	KX555512	630bp	Menglun Town, Mengla County, Yunnan Province, China
*Sinocoelotes guangxian* (Zhang et al., 2003)	KX555515	630bp	Xiaguan Town, Yunnan Province, China
*Sinocoelotes hehuaensis* sp. n.	KX555513	630bp	Hehua Village, Xiaguan Town, Yunnan Province, China
*Sinocoelotes kangdingensis* sp. n.	KX555510	630bp	Kangding County, Sichuan Province, China
*Sinocoelotes ludingensis* sp. n.	KX555509	627bp	Luding County, Sichuan Province, China
*Sinocoelotes luoshuiensis* sp. n.	KX555517	630bp	Jiangdong Village, Gudong Town, Yunnan Province, China
*Sinocoelotes mahuanggouensis* sp. n.	KX555508	630bp	Baoxing County, Sichuan Province, China
*Sinocoelotes mangbangensis* sp. n.	KX555511	630bp	Changlinggan Village, Tengchong County, Yunnan Province, China
*Sinocoelotes muliensis* sp. n.	KX555520	630bp	Muli County, Sichuan Province, China
*Sinocoelotes pseudoterrestris* (Schenkel, 1963)	KX555518	627bp	Xishan Forest Park, Yunnan Province, China
*Sinocoelotes pseudoyunnanensis* (Wang et al., 2009)	KX555519	630bp	Pianma Town, Lushui County, Yunnan Province, China
*Sinocoelotes thailandensis* (Dankittipakul & Wang, 2003)	KX555507	630bp	Jeep tract, Mae Cham District, Chiangmai Province, Thailand
*Sinocoelotes yanyuanensis* sp. n.	KX555506	630bp	Yanyuan County, Sichuan Province, China

All of the specimens (including molecular vouchers) are deposited in the Institute of Zoology, Chinese Academy of Sciences
(IZCAS) in Beijing, China.

## Systematics

### Family Agelenidae C.L. Koch, 1837 Subfamily Coelotinae F.O.P.-Cambridge, 1893

#### Genus Sinocoelotes

Taxon classificationAnimaliaAraneaeAgelenidae

Zhao & Li gen. n.

http://zoobank.org/1AD20278-53C5-40CE-90A8-A888588CE81D

##### Type species.

*Sinocoelotes hehuaensis* sp. n.

##### Etymology.

The generic name is derived from its similarity to *Coelotes* and the Latin adjective Sino- for “Chinese” referring to the main distribution region of the genus. The gender is masculine.

##### Diagnosis.

**Figure 1.**
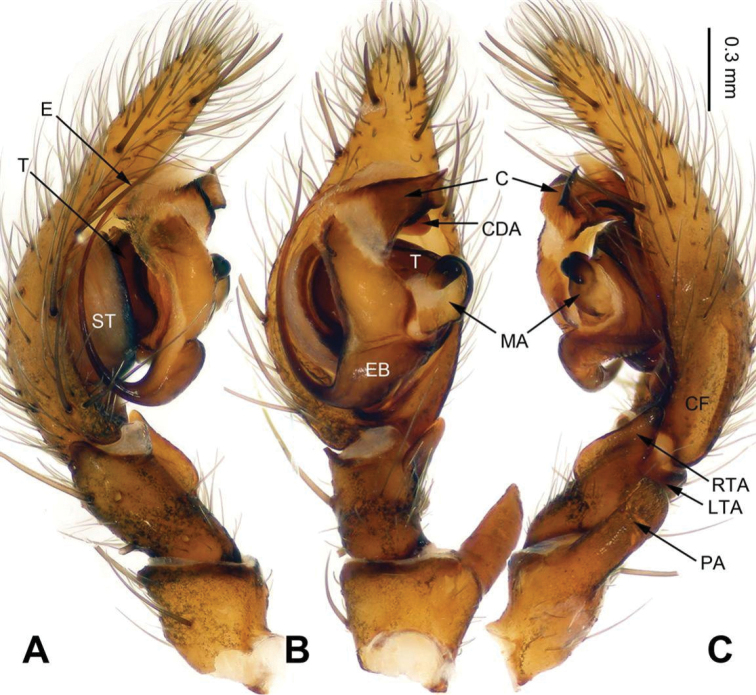
Male palp of *Coelotes pickardi tirolensis*, from Italy. **A** Prolateral **B** Ventral **C** Retrolateral. Scale bar: equal for **A, B** and **C**.

**Figure 2.**
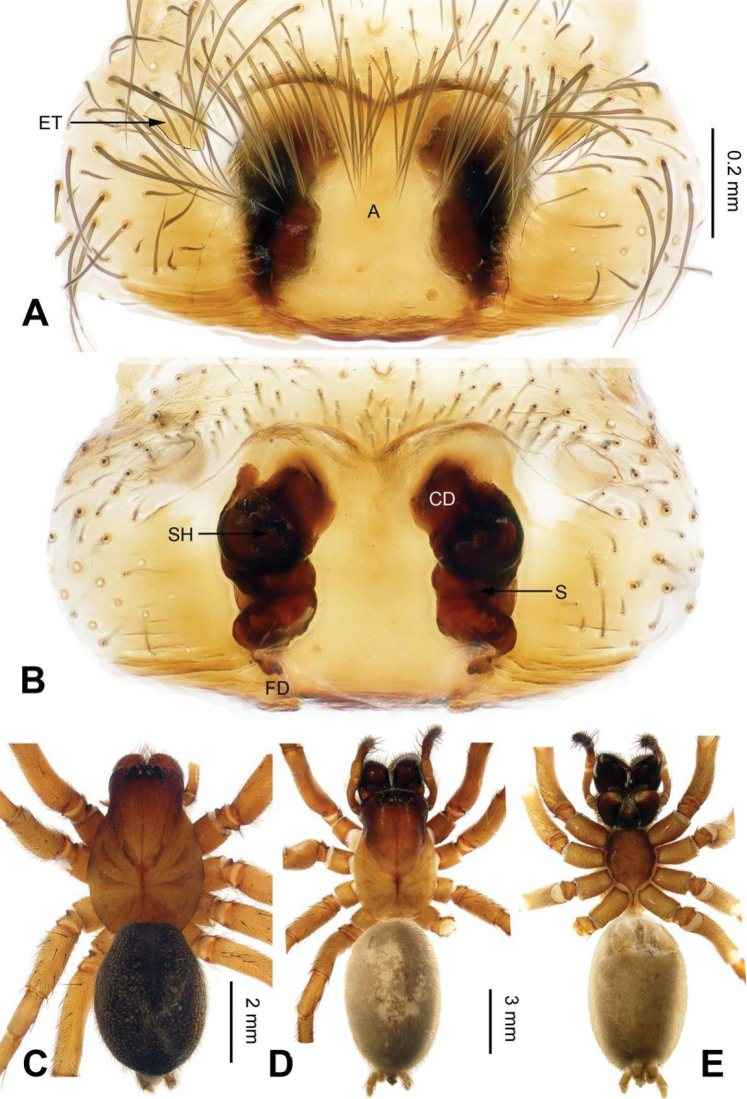
Epigyne and habitus of *Coelotes pickardi tirolensis*, from Italy. **A** Epigyne, ventral **B** Vulva, dorsal **C** Male habitus, dorsal **D** Female habitus, dorsal **E** Female habitus, ventral. Scale bars: equal for **A** and **B**; equal for **D** and **E**.

*Sinocoelotes* gen. n. is similar to *Coelotes*. Males of new genus can be distinguished from those of *Coelotes* by the longer and slenderer conductor (about 1/2 length of cymbium, while in *Coelotes* the conductor is broad, less than 1/4 length of cymbium in length, and with blunt tip, see Fig. 1), the shorter and truncated patellar apophysis with a blackened apex, while in *Coelotes* the patellar apophysis is longer than patella, and usually with a ventral part extending longer than dorsal part (see Fig. 1), the short LTA (less than 1/6 length of RTA) (cf. Figs 7A–C and 1A–C). Females of *Sinocoelotes* gen. n. can be distinguished from those of *Coelotes* by the longer copulatory ducts, about 1/2 length of vulva (while in *Coelotes* they are very short or even absent, Fig. 2A–B), the shorter spermathecae (about 1/2 length of epigyne), which can be divided into two parts: anterior part and posterior part (while *Coelotes* has spermathecae subequal to the length of epigyne, and usually S-shaped), and by the oval or finger-like spermathecal heads (while in *Coelotes* spermathecal heads are short, rounded and situated at the anterior part of epigyne) (cf. Figs 8A–B and 2A–B).

##### Description.

Small to medium-sized, with a total length of 5–14 mm, the body brown to dark brown. Carapace narrowed in ocular area, sparsely covered with black setae, and thoracic region with longitudinal fovea and radial groove; sternum usually heart-shaped. Abdomen brown to dark brown, heavily covered with short setae; dorsum with many black irregular patches and five grey chevron-like markings, the antero-median part with one cardiac pattern, and posterior part with dark maculation. Chelicerae with 3 promarginal and 3 or 4 retromarginal teeth. Male palp with one patellar apophysis and two tibial apophyses (RTA and LTA), the patellar apophysis broad and long, its apex blackened and truncated; RTA long and broad, extending beyond distal margin of tibia, subequal to the length of tibia, and with blunt tip; LTA short; cymbium crescent-shaped, the tip long, about 1/3 length of cymbium; cymbial furrow short, less than 1/4 of the cymbium; tegulum slender, and visible part very small; conductor slender, tapered, and it’s apex not close to the tegulum; median apophysis present, spoon-like; dorsal conductor apophysis well developed. Epigyne with large atrium (occupying about 1/4 of epigynal plate square); epigynal teeth present, long or short, located medially in comparison to epigynal plate height or antero-medially; spermathecae usually long and convoluted, subdivided in 2 parts: anterior and posterior; anterior part of spermathecae broad, posterior part thinner and strongly convoluted, anterior part usually larger than posterior part; spermathecal heads located at the border between anterior part of spermathecae and copulatory ducts; copulatory ducts broad, arc-shaped, situated anteriorly, connected to each other at basal part, and separated about its length at terminal part.

##### Comments.

In addition to morphological study, we analyzed the relationships of coelotine spiders based on molecular data (8 genes, ~ 6.5 kb) on 18 genera and 286 coelotine species. The molecular analyses (in progress) support *Sinocoelotes* gen. n. as monophyletic.

##### Distribution.

So far the genus is known only from China and Thailand (Fig. 21).

#### Sinocoelotes acicularis

Taxon classificationAnimaliaAraneaeAgelenidae

(Wang, Griswold & Ubick, 2009) comb. n.

Figs 3 , 21

Coelotes acicularis Wang et al. 2009: 4, figs 1–9 (♂♀, from Baoshan, Yunnan, China, in HNU and CAS, not examined).

##### Material examined.

1♀: China: Yunnan Province: Nujiang Lisu Autonomous Prefecture: Lushui County, road from Liuku to Pianma Town, N26°00'09", E98°39'33", 2422 m, 7.XII.2013, Y. Li and J. Liu.

##### Diagnosis.

**Figure 3.**
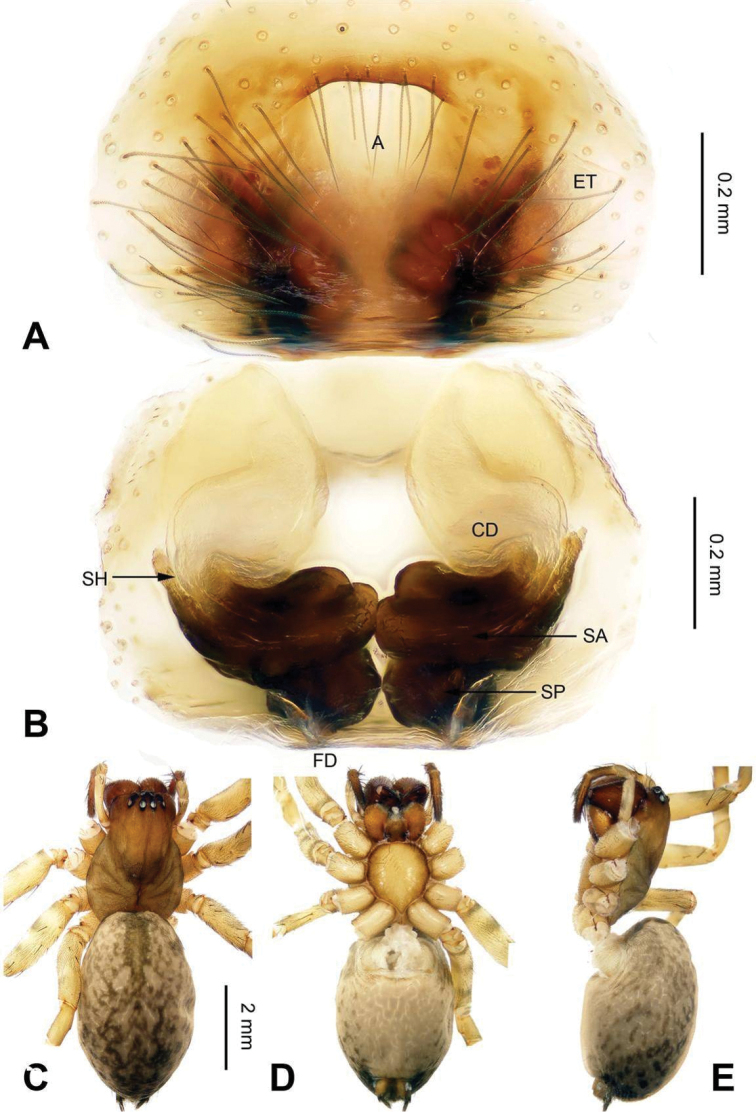
Epigyne and habitus of *Sinocoelotes acicularis*. **A** Epigyne, ventral **B** Vulva, dorsal **C** Female habitus, dorsal **D** Female habitus, ventral **E** Female habitus, lateral. Scale bars: equal for **C, D** and **E**.

The female is similar to *Sinocoelotes hehuaensis* sp. n., but can be easily distinguished from it by the longer epigynal teeth (three times longer than in *Sinocoelotes hehuaensis* sp. n.), the different shape of atrium (anterior part much broader than posterior part in this species, but inverted U-shaped in *Sinocoelotes hehuaensis* sp. n.), and the broader and membranous copulatory ducts (which are slender and sclerotized in *Sinocoelotes hehuaensis* sp. n.) (cf. Figs 3A–B and 8A–B).

##### Description.

Described by Wang et al. (2009).

##### Comments.

The species shares a combination of somatic morphology characters with *Sinocoelotes hehuaensis* sp. n., and therefore we transfer it to *Sinocoelotes* gen. n. The molecular analysis supports this transfer.

##### Distribution.

China (Yunnan) (Fig. 21).

#### Sinocoelotes cangshanensis

Taxon classificationAnimaliaAraneaeAgelenidae

Zhao & Li sp. n.

http://zoobank.org/0E3AEB72-937A-4AF6-8238-2AFF88E18C3F

Figs 4 , 21

##### Type material.

**Holotype** ♀: China: Yunnan Province: Dali Bai Autonomous Prefecture: Xiaguan Town, Hehua Village, Cangshan Mountain, Baolinjing valley, N25°36'27", E100°11'18", 2307 m, 20.XI.2013, Y. Li and J. Liu.

##### Etymology.

The specific name refers to the type locality; adjective.

##### Diagnosis.

**Figure 4.**
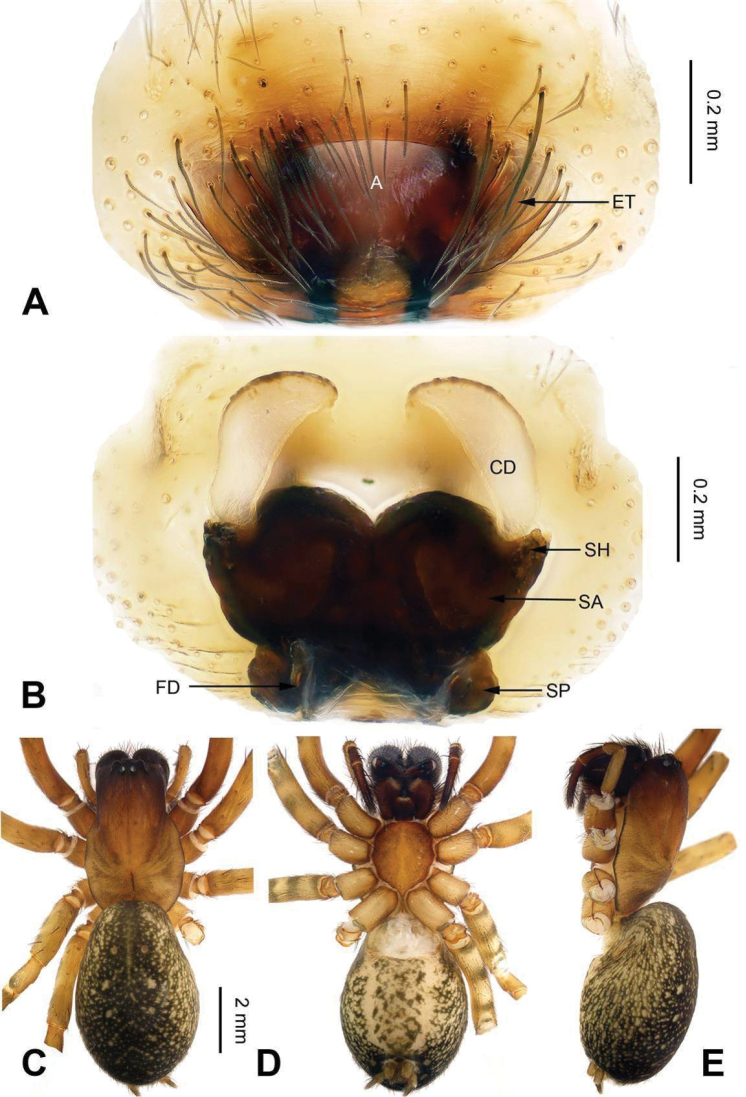
Epigyne and habitus of *Sinocoelotes cangshanensis* sp. n., holotype. **A** Epigyne, ventral **B** Vulva, dorsal **C** Female habitus, dorsal **D** Female habitus, ventral **E** Female habitus, lateral. Scale bars: equal for **C, D** and **E**.

The female can be easily distinguished from other *Sinocoelotes* gen. n. species by the long and broad epigynal teeth (subequal to the atrial length), the broad anterior part of spermathecae (occupying 1/4 of epigyne plate square, and about five times of the posterior part of spermathecae in this species, but occupying less than 1/5 epigyne plate square in other species), anterior part of spermathecae touching each other (only part of SA touching each other in *Sinocoelotes hehuaensis* sp. n. and *Sinocoelotes mangbangensis* sp. n.; part of SP touching each other in *Sinocoelotes luoshuiensis* sp. n. and *Sinocoelotes pseudoterrestris* comb. n.; separated from each other in other species), and the short, laterally located spermathecal heads (laterally located but long in *Sinocoelotes acicularis* comb. n., *Sinocoelotes kangdingensis* sp. n. and *Sinocoelotes mahuanggouensis* sp. n.; medially located in other species) (Fig. 4A–B).

##### Description.

**Female.** Total length 9.82. Carapace 4.50 long, 3.04 wide. Abdomen 5.32 long, 3.76 wide. Eye sizes and interdistances: AME 0.16, ALE 0.22, PME 0.18, PLE 0.21; AME-AME 0.08, AME-ALE 0.04, PME-PME 0.13, PME-PLE 0.22. Leg measurements: I 11.50 (3.35, 3.45, 2.75, 1.95); II 12.24 (3.20, 4.16, 3.12, 1.76); III 10.84 (2.88 3.28, 2.88, 1.80); IV 14.57 (3.92, 4.10, 4.23, 2.32). Chelicerae with four retromarginal teeth. Epigyne: atrium small, occupying 1/6 of epigynal plate square, narrowing at the middle part; teeth long, broad, located anteriorly, close to atrial anterior margin, and their length subequal to atrial length, width subequal to atrium width; spermathecae contiguous with each other, anterior part of spermathecae broad; posterior part of spermathecae about four times thinner than the anterior part; spermathecal heads small, located laterally; copulatory openings hidden in anterior part of atrium; copulatory ducts membranous, anterior parts separated from each other by 0.3 length, posterior part separated by approximately 1.5 times length, copulatory duct first goes anteriorly, and then posteriorly (Fig. 4A–B).

**Male.** Unknown.

##### Distribution.

Known only from the type locality (Fig. 21).

#### Sinocoelotes forficatus

Taxon classificationAnimaliaAraneaeAgelenidae

(Liu & Li, 2010) comb. n.

Figs 5 , 21

Coelotes forficatus Liu and Li 2010: 2, figs 1A–B, 2A–C, 3A–B, 4A–B, 5A–C (♂ holotype and ♂♀ paratypes from Xishuangbanna, Yunnan, China, in IZCAS, not examined).

##### Material examined.

1♀: China: Yunnan Province: Xishuangbanna Dai Autonomous Prefecture: Mengla County, Menglun Town, Xishuangbanna Nature Reserve, N21°37'55", E101°12'25", 665 m, 3.VII.2013, Q. Zhao and Z. Chen.

##### Diagnosis.

**Figure 5.**
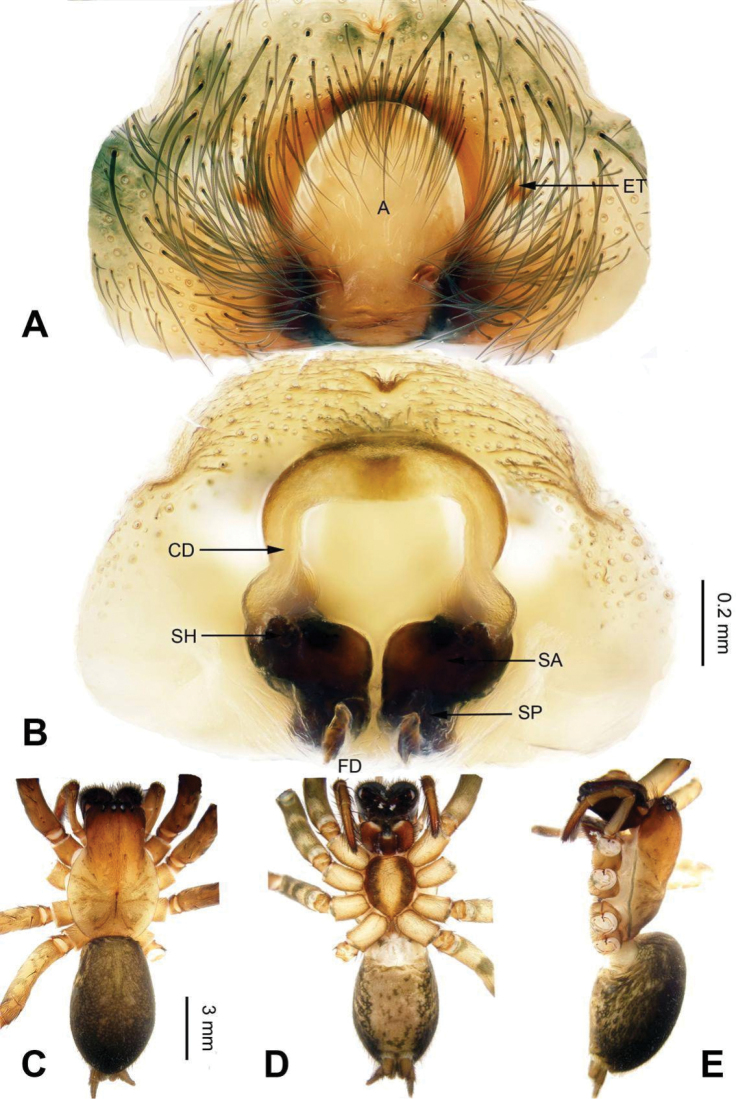
Epigyne and habitus of *Sinocoelotes forficatus*. **A** Epigyne, ventral **B** Vulva, dorsal **C** Female habitus, dorsal **D** Female habitus, ventral **E** Female habitus, lateral. Scale bars: equal for **A** and **B**; equal for **C, D** and **E**.

The female is similar to *Sinocoelotes hehuaensis* sp. n., but can be easily distinguished from it by the longer and slenderer epigynal teeth (twice as long as in *Sinocoelotes hehuaensis* sp. n.), the broader, shorter and laterally originating spermathecal heads (twice as long as *Sinocoelotes forficatus* and medially originating in *Sinocoelotes hehuaensis* sp. n.), and the slenderer, longer and inverted U-shaped copulatory ducts (cf. Figs 5A–B and 8A–B).

##### Comments.

The species shares a combination of somatic morphology characters with *Sinocoelotes hehuaensis* sp. n., and therefore we assigned it to *Sinocoelotes* gen. n. The molecular analysis supports this transfer.

##### Description.

Described by Liu and Li (2010).

##### Distribution.

China (Yunnan) (Fig. 21).

#### Sinocoelotes guangxian

Taxon classificationAnimaliaAraneaeAgelenidae

(Zhang, Yang, Zhu & Song, 2003) comb. n.

Figs 6 , 21

Coelotes guangxian Zhang et al. 2003: 79, figs 1–5 (♂ holotype and ♂♀ paratypes from Dali, Yunnan, China, in MHBU, not examined).

##### Material examined.

1♀: China: Yunnan Province: Dali Bai Autonomous Prefecture: Xiaguan Town, the south shore of Erhai Lake, Tuanshan Park, N25°36'27", E100°14'39", 1992 m, 19.XI.2013, Y. Li and J. Liu.

##### Diagnosis.

**Figure 6.**
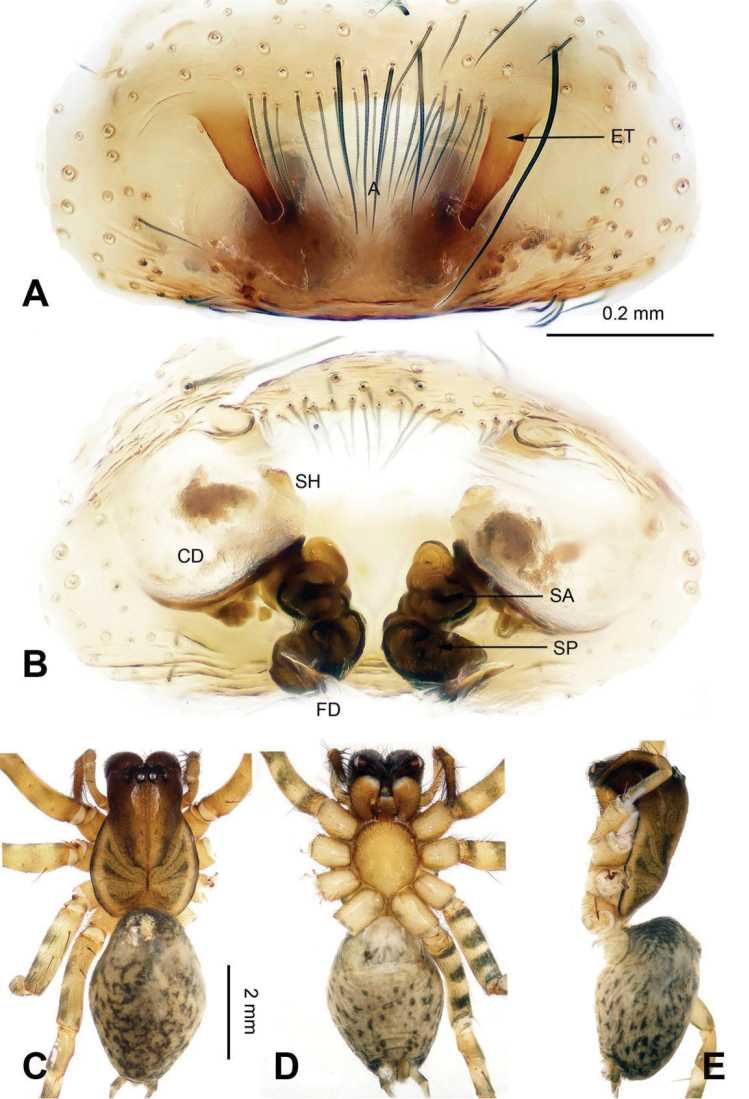
Epigyne and habitus of *Sinocoelotes guangxian*. **A** Epigyne, ventral **B** Vulva, dorsal **C** Female habitus, dorsal **D** Female habitus, ventral **E** Female habitus, lateral. Scale bars: equal for **A** and **B**; equal for **C, D** and **E**.

The female can be easily distinguished from all other *Sinocoelotes* gen. n. species by the broad atrium, the long, with blunt tip and anteriorly situated epigynal teeth (long, anteriorly situated, but with pointed tip in *Sinocoelotes kangdingensis* sp. n., *Sinocoelotes ludingensis* sp. n. and *Sinocoelotes luoshuiensis* sp. n.; long, with blunt tip, but not anteriorly located in *Sinocoelotes acicularis* comb. n. and *Sinocoelotes cangshanensis* sp. n.; short, less than 1/2 length of *Sinocoelotes guangxian* comb. n. in other species), the short spermathecae (anterior part is smaller than posterior part), and the broad copulatory ducts (occupying 1/2 of epigynal plate) (Fig. 6A–B).

##### Description.

See Zhang et al. (2003).

##### Comments.

The species shares a combination of somatic morphology characters with *Sinocoelotes hehuaensis* sp. n., and therefore was assigned to *Sinocoelotes* gen. n. The molecular analysis supports the transfer.

##### Distribution.

China (Yunnan) (Fig. 21).

#### Sinocoelotes hehuaensis

Taxon classificationAnimaliaAraneaeAgelenidae

Zhao & Li sp. n.

http://zoobank.org/D6B4F7D0-1DF0-4944-B82F-1FE872142A38

Figs 7 , 8 , 21

##### Type material.

**Holotype** ♂: China: Yunnan Province: Dali Bai Autonomous Prefecture: Xiaguan Town, Hehua Village, Cangshan Mountain, Baolinjing Valley, N25°36'27", E100°11'18", 2307 m, 20.XI.2013, Y. Li and J. Liu. **Paratype**: 1♀, same data as holotype.

##### Etymology.

The specific name refers to the type locality; adjective.

##### Diagnosis.

**Figure 7.**
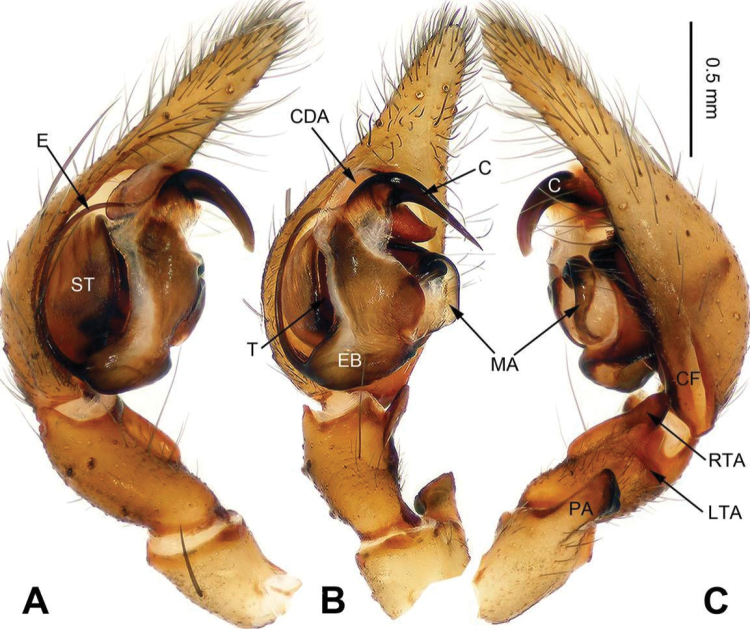
Male palp of *Sinocoelotes hehuaensis* sp. n., holotype. **A** Prolateral **B** Ventral **C** Retrolateral. Scale bar: equal for **A, B** and **C**.

**Figure 8.**
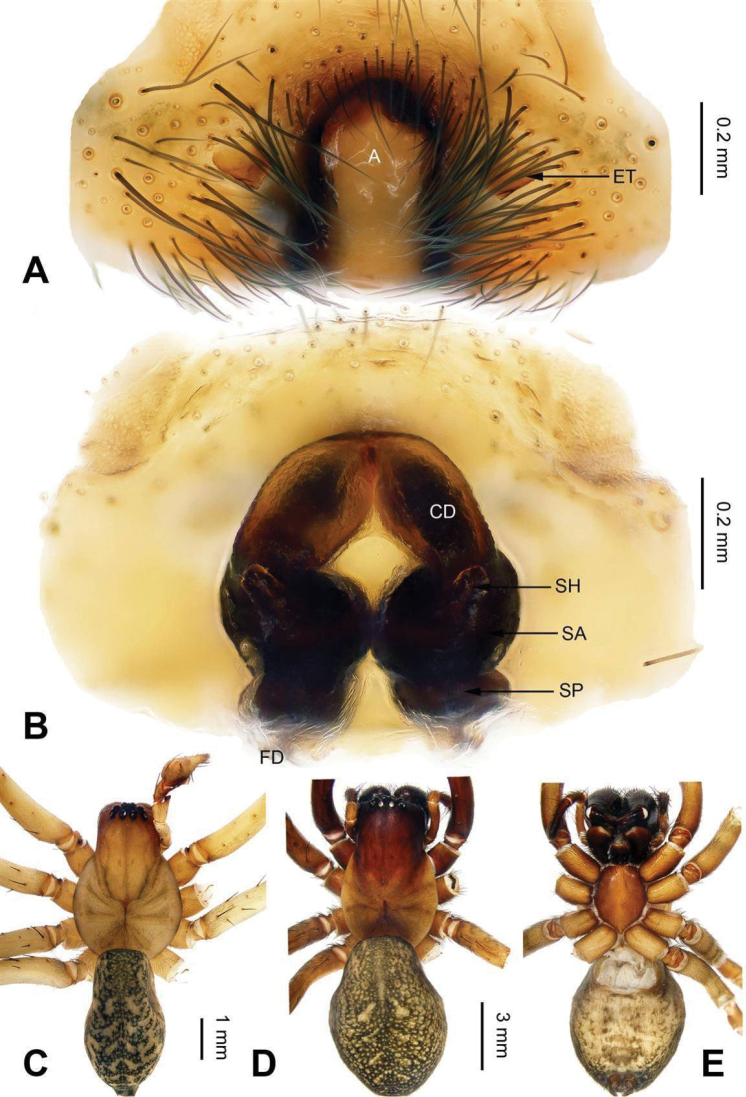
Epigyne and habitus of *Sinocoelotes hehuaensis* sp. n., holotype and paratype. **A** Epigyne, ventral **B** Vulva, dorsal **C** Male habitus, dorsal **D** Female habitus, dorsal **E** Female habitus, ventral. Scale bars: equal for **D** and **E**.

The male can be easily distinguished from other *Sinocoelotes* gen. n. species by the longer peg-shaped conductor (about 1/2 length of cymbium; less than 1/3 length of cymbium in *Sinocoelotes ludingensis* sp. n., *Sinocoelotes thailandensis*; bended in *Sinocoelotes yanyuanensis* sp. n.), the longer patellar apophysis (subequal to the length of patella in *Sinocoelotes hehuaensis* sp. n., shorter than the length of patella in other species), the larger and subtriangular dorsal conductor apophysis (large, but with blunt tip in *Sinocoelotes thailandensis*; less than 1/3 length and 1/2 width of *Sinocoelotes hehuaensis* in other species) (cf. Figs 7A–C, 10A–C, 18A–C and 20A–C). The female is similar to *Sinocoelotes cangshanensis* sp. n. but can be distinguished from it by the shorter epigynal teeth (less than 1/3 length of the teeth in *Sinocoelotes cangshanensis* sp. n.), the broader copulatory ducts (two times wider than in *Sinocoelotes cangshanensis* sp. n.), and the longer spermathecal heads (twice as long as in *Sinocoelotes cangshanensis* sp. n.) (cf. Figs 8A–B; and 4A–B).

##### Description.

**Male.** Total length 7.04. Carapace 3.60 long, 2.76 wide. Abdomen 3.44 long, 2.00 wide. Eye sizes and interdistances: AME 0.16, ALE 0.20, PME 0.19, PLE 0.17; AME-AME 0.09, AME-ALE 0.03, PME-PME 0.10, PME-PLE 0.13. Leg measurements: I 14.40 (3.92, 4.48, 3.64, 2.36); II 12.60 (3.48, 4.00, 3.20, 1.92); III 11.33 (3.28, 3.40, 3.02, 1.63); IV 15.23 (4.10, 4.50, 4.48, 2.15). Chelicerae with four retromarginal teeth. Palp: patellar apophysis long, subequal to the length of patella; RTA broad, extending beyond the tibia; LTA short, less than 1/5 length of RTA; cymbial furrow short, about 1/6 length of cymbium; conductor long, slender, peg-shaped in ventral view, subequal to 1/2 length of cymbium; dorsal conductor apophysis broad, the visible part (between conductor and tegulum) subtriangular; embolus beginning at seven o’clock position (Fig. 7A–C).

**Female.** Total length 13.20. Carapace 6.02 long, 4.49 wide. Abdomen 7.18 long, 5.26 wide. Eye sizes and interdistances: AME 0.17, ALE 0.29, PME 0.23, PLE 0.27; AME-AME 0.15, AME-ALE 0.04, PME-PME 0.24, PME-PLE 0.29. Leg measurements: I 16.83 (4.49, 5.76, 4.04, 2.54); II 15.13 (4.36, 5.06, 3.53, 2.18); III 13.99 (3.92, 4.49, 3.52, 2.06); IV 17.69 (4.95, 5.78, 4.68, 2.28). Chelicerae as in male. Epigyne: teeth short, subtriangular, located at posterior 1/2 of epigyne; copulatory ducts broad, long, sclerotized, anterior part connected to each other, and it about half of vulval length, almost as wide as spermathecae; spermathecae short and convoluted; anterior part touching each other, posterior part about 1/3 length of anterior part; spermathecal heads long, stick-shaped, twice longer than their width, originating from middle of anterior spermathecae (Fig. 8A–B).

##### Distribution.

Known only from the type locality (Fig. 21).

#### Sinocoelotes kangdingensis

Taxon classificationAnimaliaAraneaeAgelenidae

Zhao & Li sp. n.

http://zoobank.org/289D50FA-40AD-469B-B3E8-EEFCE4BBE2F1

Figs 9 , 21

##### Type material.

**Holotype** ♀: China: Sichuan Province: Garzê Tibetan Autonomous Prefecture: Kangding County, foothills of Paoma Mountain, N30°02'50", E101°58'08", 2900 m, 12.X.2005, X. Zhang and X. Xu.

##### Etymology.

The specific name refers to the type locality; adjective.

##### Diagnosis.

**Figure 9.**
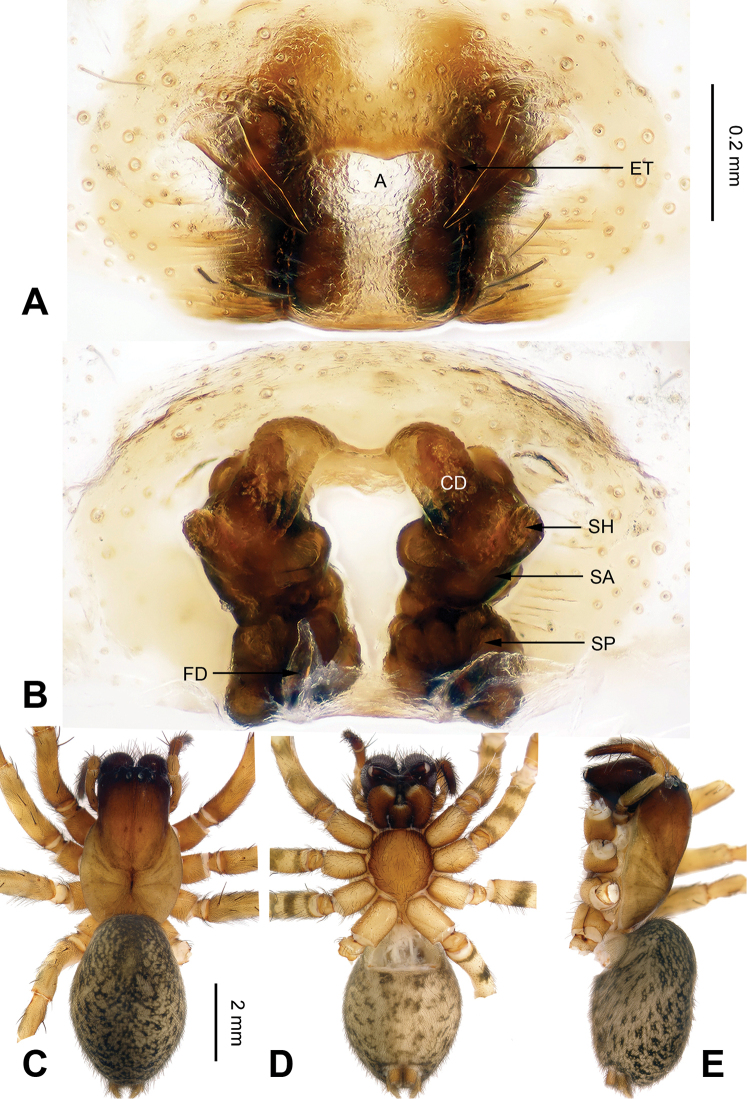
Epigyne and habitus of *Sinocoelotes kangdingensis* sp. n., holotype. **A** Epigyne, ventral **B** Vulva, dorsal **C** Female habitus, dorsal **D** Female habitus, ventral **E** Female habitus, lateral. Scale bars: equal for **A, B**; equal for **C, D** and **E**.

The female is similar to that of *Sinocoelotes cangshanensis* sp. n., but can be distinguished from it by the slenderer epigynal teeth (about 1/2 width of *Sinocoelotes cangshanensis* sp. n.), the broader SA, the thinner PA, the ratio between two parts of spermathecae (the PA subequal to the SA in *Sinocoelotes kangdingensis* sp. n., but PA just about 1/4 of the SA in *Sinocoelotes cangshanensis* sp. n.) (cf. Figs 9A–B and 2A–B), the well sclerotized copulatory ducts (Fig. 9A–B).

##### Description.

**Female.** Total length 8.20. Carapace 3.80 long, 2.65 wide. Abdomen 4.40 long, 2.95 wide. Eye sizes and interdistances: AME 0.14, ALE 0.20, PME 0.15, PLE 0.19; AME-AME 0.10, AME-ALE 0.05, PME-PME 0.11, PME-PLE 0.17. Leg measurements: I 9.72 (2.72, 3.28, 2.28, 1.44); II 8.69 (2.50, 2.81, 2.13, 1.25); III 8.06 (2.25, 2.59, 2.07, 1.15); IV 10.76 (2.96, 3.52, 2.96, 1.32). Chelicerae with three retromarginal teeth. Epigyne: atrium small, almost rectanguala, posterior part slightly wider than anterior part, about 1/3 width and 1/2 length of epigyne, and it occupying about 1/5 of epigyne plate square; teeth broad, long, subequal to the length of atrium, located anteriorly; spermathecae separated from each other, anterior part by spermathecal width, and posterior part by 1/4 spermathecal width, posterior part subequal to the anterior part; spermathecal heads broad, short, located laterally; copulatory ducts short, slightly sclerotized, semitransparent, terminal parts leading to copulatory opening almost reduced (Fig. 9A–B).

**Male.** Unknown.

##### Distribution.

Known only from the type locality (Fig. 21).

#### Sinocoelotes ludingensis

Taxon classificationAnimaliaAraneaeAgelenidae

Zhao & Li sp. n.

http://zoobank.org/A7E9C92C-652C-46B6-B92D-A1054355BB2C

Figs 10 , 11 , 21

##### Type material.

**Holotype** ♂: China: Sichuan Province: Garzê Tibetan Autonomous Prefecture: Luding County, the road from Moxi Town to Yajiageng, N29°46'31", E102°03'34", 2412 m, 10.X.2005, X. Zhang and X. Xu. **Paratype**: 1♀, same data as holotype.

##### Etymology.

The specific name refers to the type locality; adjective.

##### Diagnosis.

**Figure 10.**
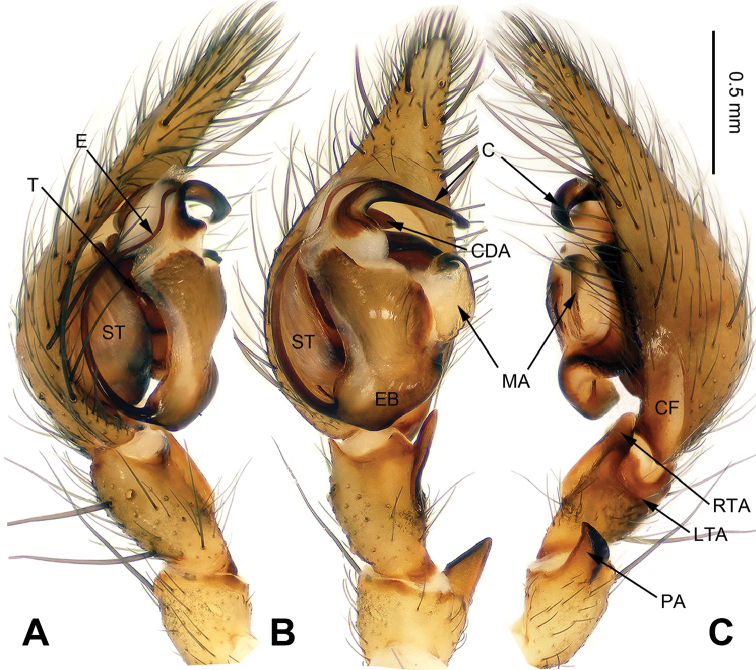
Male palp of *Sinocoelotes ludingensis* sp. n., holotype. **A** Prolateral **B** Ventral **C** Retrolateral. Scale bar: equal for **A, B** and **C**.

**Figure 11.**
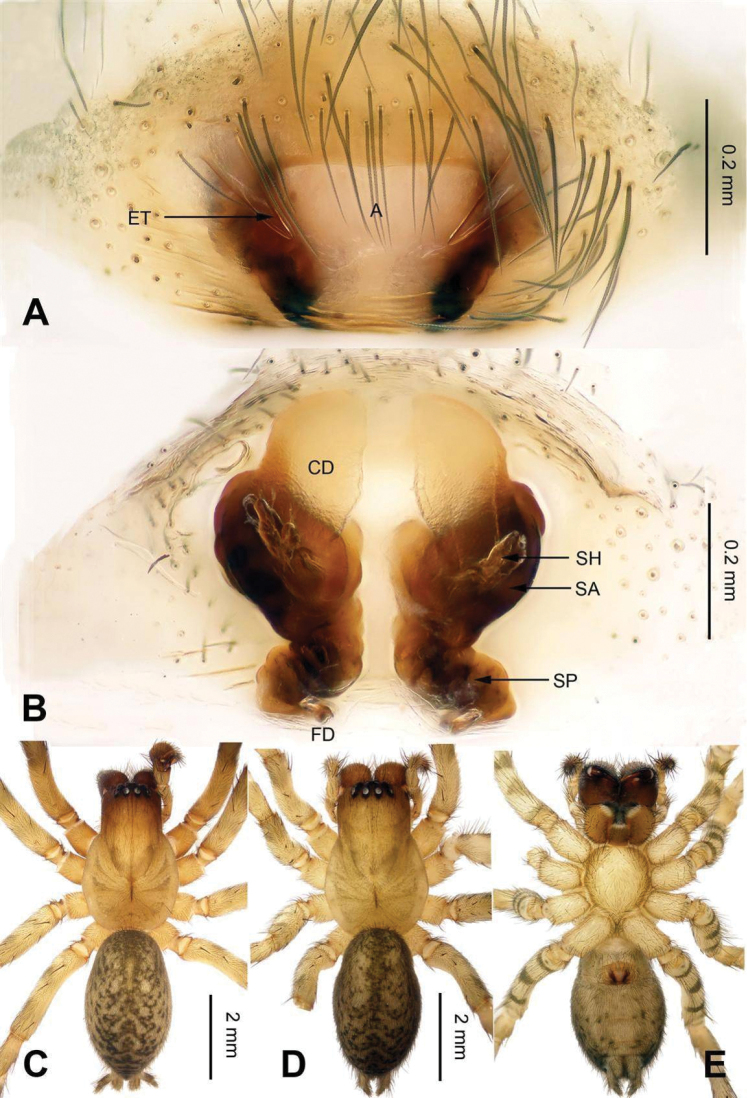
Epigyne and habitus of *Sinocoelotes ludingensis* sp. n., holotype and paratype. **A** Epigyne, ventral **B** Vulva, dorsal **C** Male habitus, dorsal **D** Female habitus, dorsal **E** Female habitus, ventral. Scale bars: equal for **D** and **E**.

The male is similar to that of *Sinocoelotes hehuaensis* sp. n., but can be distinguished from it by the slenderer conductor, with the hook-like apex (conductor peg-shaped in *Sinocoelotes hehuaensis* sp. n.), the smaller dorsal conductor apophysis (about 1/2 width and 1/3 length of *Sinocoelotes hehuaensis* sp. n.) (cf. Figs 10A–C and 7A–C). The female is similar to that of *Sinocoelotes kangdingensis* sp. n., but can be distinguished from it by the shape of atrium, anterior part wider than posterior part in *Sinocoelotes ludingensis* sp. n. (anterior part narrower than posterior part in *Sinocoelotes kangdingensis* sp. n.), the broader copulatory ducts, the longer (twice as long as *Sinocoelotes kangdingensis* sp. n.) and medially originating spermathecal heads (laterally originating in *Sinocoelotes kangdingensis* sp. n.) (cf. Figs 11A–B and 9A–B).

##### Description.

**Male.** Total length 7.12. Carapace 3.40 long, 2.40 wide. Abdomen 3.72 long, 2.20 wide. Eye sizes and interdistances: AME 0.14, ALE 0.20, PME 0.19, PLE 0.16; AME-AME 0.09, AME-ALE 0.03, PME-PME 0.09, PME-PLE 0.12. Leg measurements: I 11.04 (3.08, 3.48, 2.80, 1.68); II 9.66 (2.82, 3.13, 2.23, 1.48); III 8.82 (2.60 2.50, 2.47, 1.25); IV 12.19 (3.36, 3.76, 3.48, 1.59). Chelicerae with four retromarginal teeth. Palp: patellar apophysis short, about 1/2 of patella; RTA broad and long, subequal to the length of tibia; LTA short, less than 1/6 length of RTA; cymbial furrow short, about 1/5 length of cymbium; conductor long, slender, and apex hook-like; median apophysis spoon-like; dorsal conductor apophysis broad, the visible part (between conductor and tegulum) finger-like; embolus beginning at seven o’clock position (Fig. 10A–C).

**Female.** Total length 6.76. Carapace 3.44 long, 2.36 wide. Abdomen 3.32 long, 2.00 wide. Eye sizes and interdistances: AME 0.14, ALE 0.21, PME 0.14, PLE 0.16; AME-AME 0.09, AME-ALE 0.04, PME-PME 0.13, PME-PLE 0.16. Leg measurements: I 8.90 (2.52, 2.95, 2.08, 1.35); II 7.81 (2.28, 2.50, 1.81, 1.22); III 7.16 (2.03, 2.31, 1.84, 0.98); IV 9.66 (2.69, 3.06, 2.66, 1.25). Chelicerae as in male. Epigyne: atrium, trapezoidal, occupying 1/4 of epigynal plate square, narrowing at the posterior part; teeth long, located anterior-laterally, subequal to the atrial length; copulatory ducts membranous, semitransparent, parallel to each other, wider than basal part of spermathecae; spermathecae separated from each other by spermathecal heads’ width, basal part of spermathecae about 1/2 thinner than anterior part; spermathecal heads long, located at mid-anterior of spermathecae (Fig. 11A–B).

##### Distribution.

Known only from the type locality (Fig. 21).

#### Sinocoelotes luoshuiensis

Taxon classificationAnimaliaAraneaeAgelenidae

Zhao & Li sp. n.

http://zoobank.org/61671F60-C3D0-4A18-A2EC-3CCFB44B7B06

Figs 12 , 21

##### Type material.

**Holotype** ♀: China: Yunnan Province: Tengchong County, Gudong Town, Jiangdong Village, Jiangdong Mountain, Luoshui Cave, N24°58'06", E98°52'06", 1881 m, 26.XI.2013, Y. Li and J. Liu.

##### Etymology.

The specific name refers to the type locality; adjective.

##### Diagnosis.

**Figure 12.**
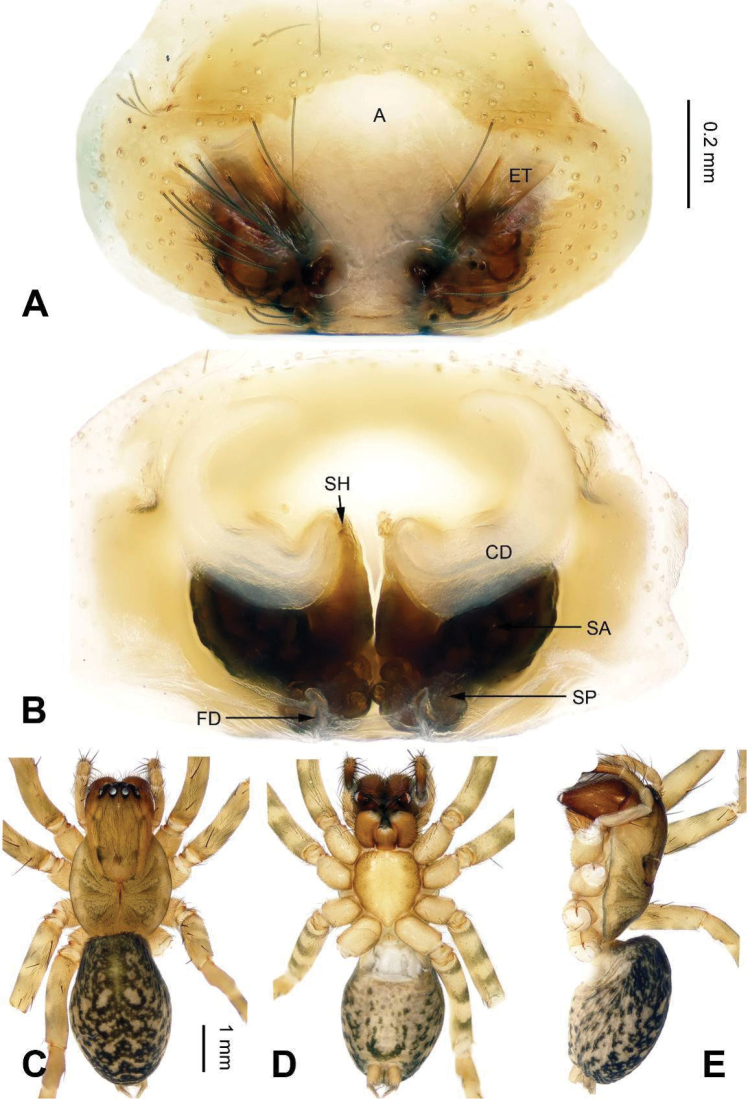
Epigyne and habitus of *Sinocoelotes luoshuiensis* sp. n., holotype. **A** Epigyne, ventral **B** Vulva, dorsal **C** Female habitus, dorsal **D** Female habitus, ventral **E** Female habitus, lateral. Scale bars: equal for **A** and **B**; equal for **C, D** and **E**.

The female of the new species has uniquely shaped epigyne and can be easily distinguished from all other *Sinocoelotes* gen. n. species by the broad atrium lacking distinct margins (with distinct anterior and lateral margins in other species), the long and sickle-shaped copulatory ducts, and copulatory ducts span wider than spermathecae, the spermathecal heads short and close to each other (close to each other but five times as long as in *Sinocoelotes luoshuiensis* sp. n in *Sinocoelotes muliensis* sp. n., and laterally originating in other species) (Fig. 12A–B).

##### Description.

**Female.** Total length 6.48. Carapace 3.28 long, 2.24 wide. Abdomen 3.20 long, 2.21 wide. Eye sizes and interdistances: AME 0.13, ALE 0.19, PME 0.16, PLE 0.17; AME-AME 0.06, AME-ALE 0.04, PME-PME 0.08, PME-PLE 0.11. Leg measurements: I 9.59 (2.66, 3.15, 2.22, 1.56); II 8.56 (2.47, 2.78, 2.01, 1.30); III 7.72 (2.15, 2.42, 1.98, 1.17); IV 10.37 (2.81, 3.28, 3.82, 1.46). Chelicerae with four retromarginal teeth. Epigyne: atrium large, about 1/3 of epigynal plate square, without sharp boundary, narrowing at posterior part; teeth long, about 1/2 length of atrium; spermathecae close to each other, posterior part about 1/5 of posterior part; spermathecal heads long, located mesally, close to each other; copulatory ducts long, hook-like (Fig. 12A–B).

**Male.** Unknown.

##### Distribution.

Known only from the type locality (Fig. 21).

#### Sinocoelotes mahuanggouensis

Taxon classificationAnimaliaAraneaeAgelenidae

Zhao & Li sp. n.

http://zoobank.org/5EC7587F-22FA-46E9-85D2-70EDBBFAAEF1

Figs 13 , 21

##### Type material.

**Holotype** ♀: China: Sichuan Province: Baoxing County, Fengtongzhai Nature Reserve, Mahuang valley, under stones, N30°49'27", E102°44'16", 2440 m, 27.IX.2005, X. Zhang and X. Xu.

##### Etymology.

The specific name refers to the type locality; adjective.

##### Diagnosis.

**Figure 13.**
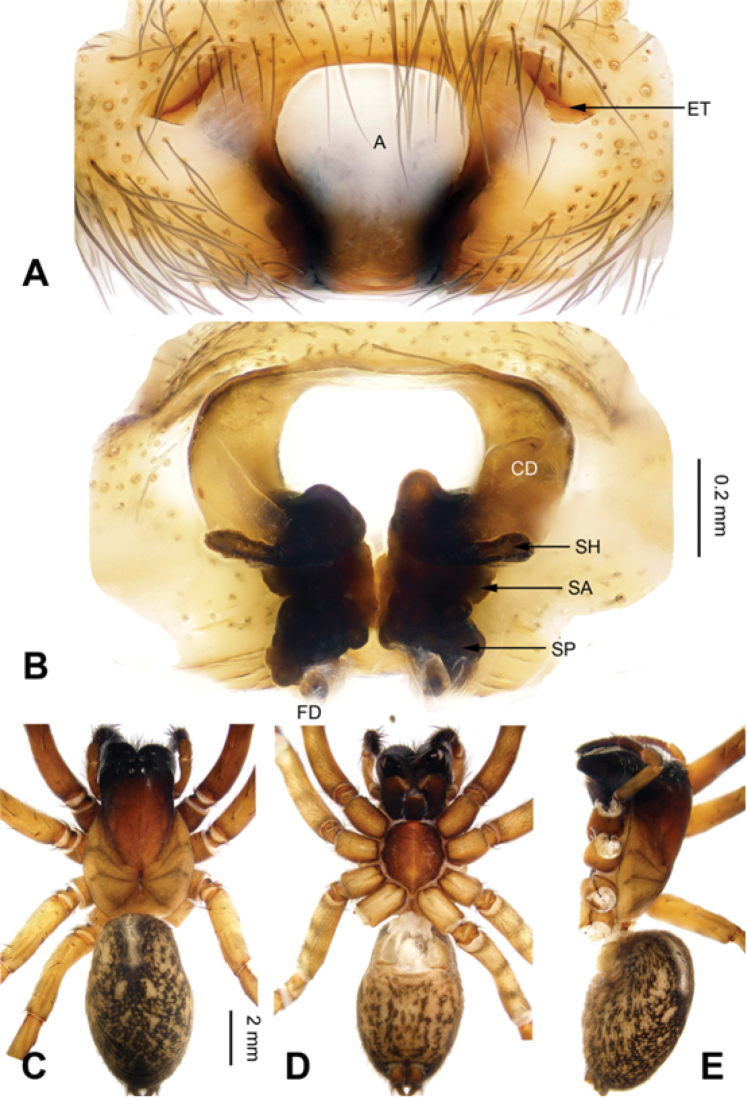
Epigyne and habitus of *Sinocoelotes mahuanggouensis* sp. n., holotype. **A** Epigyne, ventral **B** Vulva, dorsal **C** Female habitus, dorsal **D** Female habitus, ventral **E** Female habitus, lateral. Scale bars: equal for **A** and **B**; equal for **C, D** and **E**.

The female can be easily distinguished from other *Sinocoelotes* gen. n. species by the short, wider than long, triangular epigynal teeth, the pear-shaped atrium, the sickle-shaped copulatory ducts, the long and clavate spermathecal heads (Fig. 13A–B).

##### Description.

**Female.** Total length 11.80. Carapace 5.77 long, 3.97 wide. Abdomen 6.03 long, 3.85 wide. Eye sizes and interdistances: AME 0.26, ALE 0.25, PME 0.21, PLE 0.24; AME-AME 0.11, AME-ALE 0.09, PME-PME 0.23, PME-PLE 0.34. Leg measurements: I 17.56 (4.74, 5.96, 4.17, 2.69); II 15.75 (4.35, 5.19, 3.85, 2.36); III 14.69 (4.05, 4.55, 3.91, 2.18); IV 19.15 (5.13, 6.03, 5.45, 2.54). Chelicerae with three retromarginal teeth. Epigyne: atrium large, occupying 1/3 of epigynal plate square, narrowing posteriorly, pear-shaped; teeth short, wider than long, triangular in shape, located anterio-laterally, widely spaced from atrium; spermathecae close to each other, posterior (basal) part subequal to the anterior part; spermathecal heads long, clavate; copulatory ducts long, broad, crescent-shaped (Fig. 13A–B).

**Male.** Unknown.

##### Distribution.

Known only from the type locality (Fig. 21).

#### Sinocoelotes mangbangensis

Taxon classificationAnimaliaAraneaeAgelenidae

Zhao & Li sp. n.

http://zoobank.org/44304816-98E9-4CE4-A51E-5AB9D8F1DF37

Figs 14 , 21

##### Type material.

**Holotype** ♀: China: Yunnan Province: Tengchong County, Mangbang Village, N24°58'07", E98°36'48", 2032 m, 23.VI.2013, Z. Zhao and J. Liu.

##### Etymology.

The specific name refers to the type locality; adjective.

##### Diagnosis.

**Figure 14.**
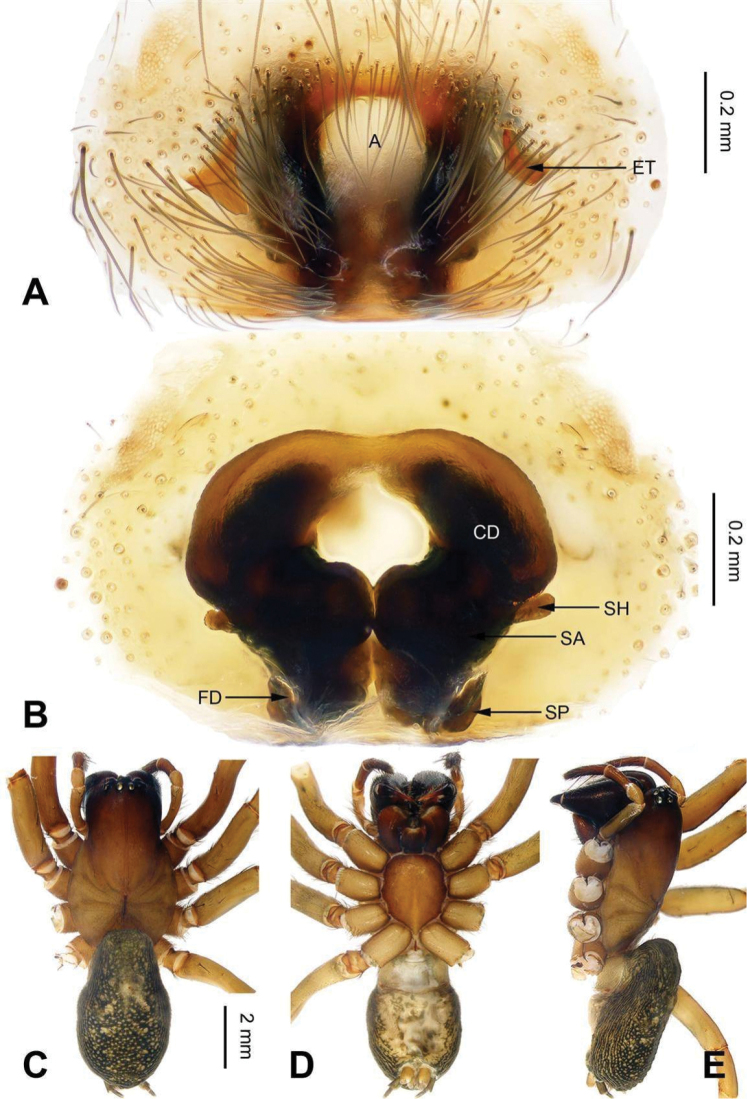
Epigyne and habitus of *Sinocoelotes mangbangensis* sp. n., holotype. **A** Epigyne, ventral **B** Vulva, dorsal **C** Female habitus, dorsal **D** Female habitus, ventral **E** Female habitus, lateral. Scale bars: equal for **C, D** and **E**.

The female is similar to that of *Sinocoelotes hehuaensis* sp. n., but can be distinguished from it by longer epigynal teeth (twice as long as in *Sinocoelotes hehuaensis* sp. n.), the broader copulatory ducts, which the posterior part separated from each other further, the slender, laterally and ventrally located spermathecal heads (dorsally situated in *Sinocoelotes hehuaensis* sp. n.) (cf. Figs 14A–B and 8A–B).

##### Description.

**Female.** Total length 10.12. Carapace 4.94 long, 3.66 wide. Abdomen 5.18 long, 3.10 wide. Eye sizes and interdistances: AME 0.21, ALE 0.29, PME 0.20, PLE 0.24; AME-AME 0.11, AME-ALE 0.05, PME-PME 0.16, PME-PLE 0.28. Leg measurements: I 14.58 (3.96, 4.95, 3.52, 2.15); II 12.81 (3.68, 4.28, 2.97, 1.88); III 11.67 (3.40, 3.72, 2.95, 1.60); IV 13.34 (4.25, 4.98, 4.22, 1.89). Sternum brown (in comparison to previous species) with light median stripe. Chelicerae with four retromarginal teeth. Epigyne: atrium small, about 1/5 of epigynal plate square, narrowing at posteriorly; teeth subtriangular, as wide as long, located laterally, near to atrial anterior margin, subequal to atrial width (narrowest part); spermathecae not spaced, posterior part thinner than posterior one; spermathecal heads long, finger-like, located laterally; copulatory ducts broad, well sclerotized, and anterior part contiguous (Fig. 14A–B).

**Male.** Unknown.

##### Distribution.

Known only from the type locality (Fig. 21).

#### Sinocoelotes muliensis

Taxon classificationAnimaliaAraneaeAgelenidae

Zhao & Li sp. n.

http://zoobank.org/8812B14A-E2BC-4054-B9D4-77BEAA37659C

Figs 15 , 21

##### Type material.

**Holotype** ♀: China: Sichuan Province: Muli County, N27°54'57", E101°16'20", 2229 m, 13.XI.2013, Y. Li and J. Liu.

##### Etymology.

The specific name refers to the type locality; adjective.

##### Diagnosis.

**Figure 15.**
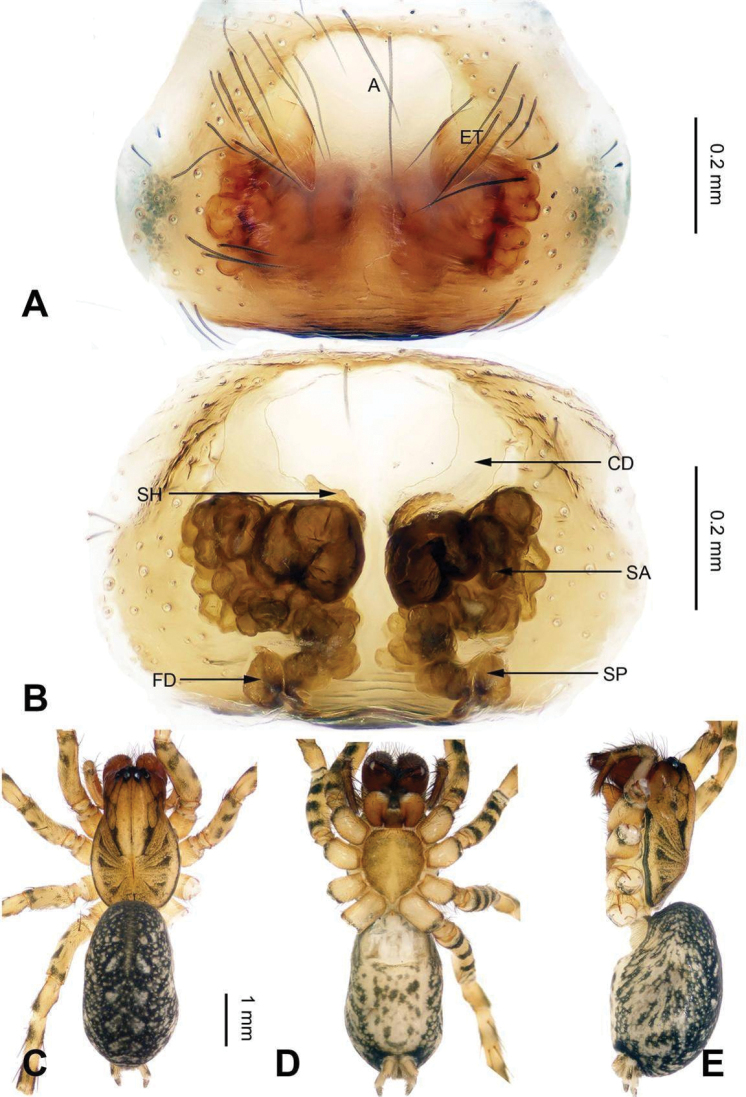
Epigyne and habitus of *Sinocoelotes muliensis* sp. n., holotype. **A** Epigyne, ventral **B** Vulva, dorsal **C** Female habitus, dorsal **D** Female habitus, ventral **E** Female habitus, lateral. Scale bars: equal for **C, D** and **E**.

The female of the new species has uniquely shaped epigyne and can be easily distinguished from all other *Sinocoelotes* gen. n. species by the anteriorly situated atrium (atrium with distinct anterior margin, but lacking distinct posterior margin, and the posterior part broader than anterior part), the teeth broad and located between two atrial lateral margins, the long and strongly twisted spermathecae, closely spaced, the slender, mesally originating spermathecal heads (which are also mesally originating in *Sinocoelotes luoshuiensis* sp. n, but are 1/4 length shorter than those in *Sinocoelotes muliensis* sp. n.; laterally originating in all other species) (Fig. 15A–B).

##### Description.

**Female.** Total length 5.76. Carapace 2.56 long, 1.72 wide. Abdomen 3.20 long, 1.87 wide. Eye sizes and interdistances: AME 0.09, ALE 0.16, PME 0.12, PLE 0.13; AME-AME 0.07, AME-ALE 0.02, PME-PME 0.08, PME-PLE 0.10. Leg measurements: I: 5.97 (1.73, 2.08, 1.38, 0.78); II: 5.40 (1.62, 1.80, 1.24, 0.74); III: 5.12 (1.50, 1.60, 1.30, 0.72); IV: 7.03 (1.94, 2.31, 1.83, 0.95). Chelicerae with 3 retromarginal teeth. Epigyne: atrium located anteriorly, occupying 1/4 of epigynal plate square, with distinct anterior margin, but lacking distinct posterior margin; teeth broad and long, located on the lateral margins of the atrium; spermathecae narrowly separated from each other, posterior part of spermathecae about 1/4 the anterior part; spermathecal heads slender and long, close to each other; copulatory ducts much thinner than anterior part of spermathecae (wider in some species), short (about 1/3 length of epigyne), membranous (Fig. 15A–B).

**Male.** Unknown.

##### Distribution.

Known only from the type locality (Fig. 21).

#### Sinocoelotes pseudoterrestris

Taxon classificationAnimaliaAraneaeAgelenidae

(Schenkel, 1963) comb. n.

Figs 16 , 21

Coelotes pseudoterrestris Schenkel 1963: 286, fig. 161 (♀ holotype from Lo Thoei Tong, Yunnan, China, in MNHP, not examined); Song et al. 1999: 378, figs 224N, 224O, 226T, 228B (♂♀, as Coelotes sacratus); Wang 2002: 52, figs 127–131 (♂♀); Wang and Jäger 2008: 2279, figs 1–2 (♂).

##### Material examined.

♀: China: Yunnan Province: Kunming City; Xishan Forest Park, the way to Longmen, in crevices on crags, N24°57'04", E102°38'18", 2437 m, 22.XII.2013, Y. Li and J. Liu.

##### Diagnosis.

**Figure 16.**
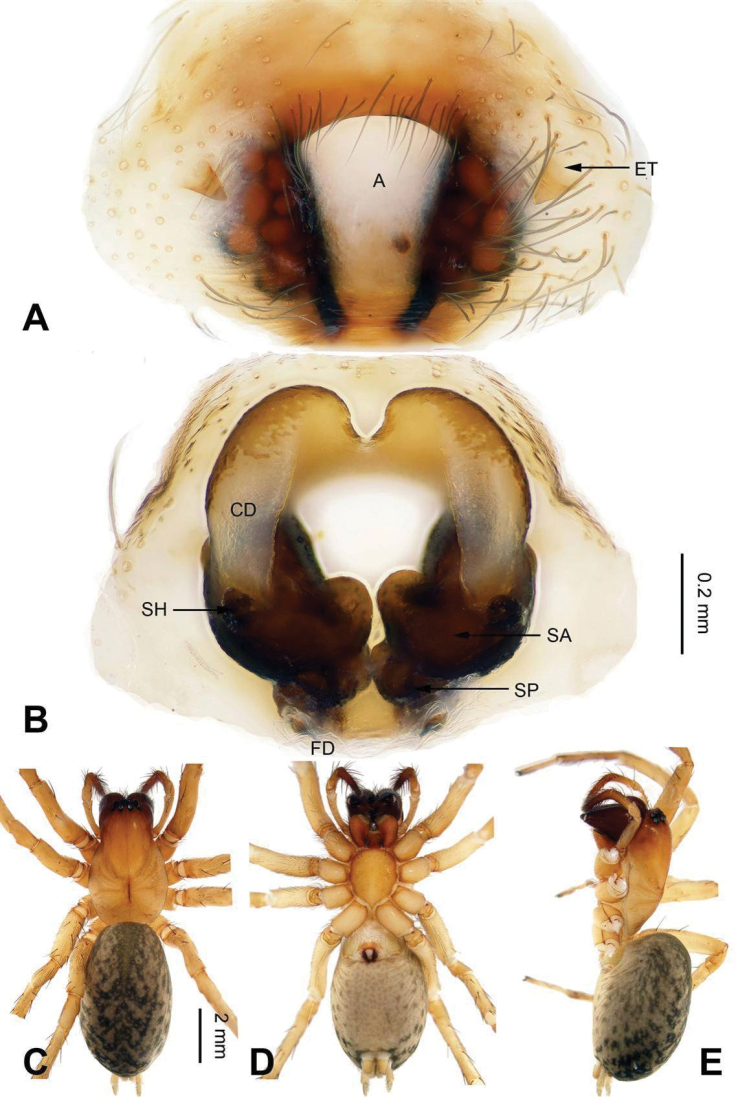
Epigyne and habitus of *Sinocoelotes pseudoterrestris*. **A** Epigyne, ventral **B** Vulva, dorsal **C** Female habitus, dorsal **D** Female habitus, ventral **E** Female habitus, lateral. Scale bars: equal for **A** and **B**; equal for **C, D** and **E**.

The female is similar to that of *Sinocoelotes mangbangensis* sp. n., but can be easily distinguished from it by the longer epigynal teeth (twice as long as in *Sinocoelotes mangbangensis* sp. n.), the smaller posterior part of spermathecae which is about 1/4 of the anterior part (the posterior part is subequal to the anterior part in *Sinocoelotes mangbangensis* sp. n.), the laterally situated spermathecal heads (ventrally situated in *Sinocoelotes mangbangensis* sp. n.), and the membranous copulatory ducts (strongly sclerotized in *Sinocoelotes mangbangensis* sp. n.) (cf. Figs 16A–B and 14 A–B).

##### Comments.

The species shares a combination of somatic morphology characters with *Sinocoelotes hehuaensis* sp. n., and therefore was assigned to *Sinocoelotes* gen. n. The molecular analysis supports the transfer.

##### Description.

Described by Wang (2002).

##### Distribution.

China (Yunnan) (Fig. 21).

#### Sinocoelotes pseudoyunnanensis

Taxon classificationAnimaliaAraneaeAgelenidae

(Wang, Griswold & Ubick, 2009) comb. n.

Figs 17 , 21

Coelotes pseudoyunnanensis Wang et al. 2009: 19, figs 88–96 (♂ holotype and ♂♀ paratypes from Nujiang, Yunnan, China, in HNU and CAS, not examined).

##### Material examined.

♂: China: Yunnan Province: Nujiang Lisu Autonomous Prefecture: Lushui County, Pianma Town, Gaoligong Mountain, N25°58'22", E98°41'02", 3133 m, 8.XII.2013, Y. Li and J. Liu.

##### Diagnosis.

**Figure 17.**
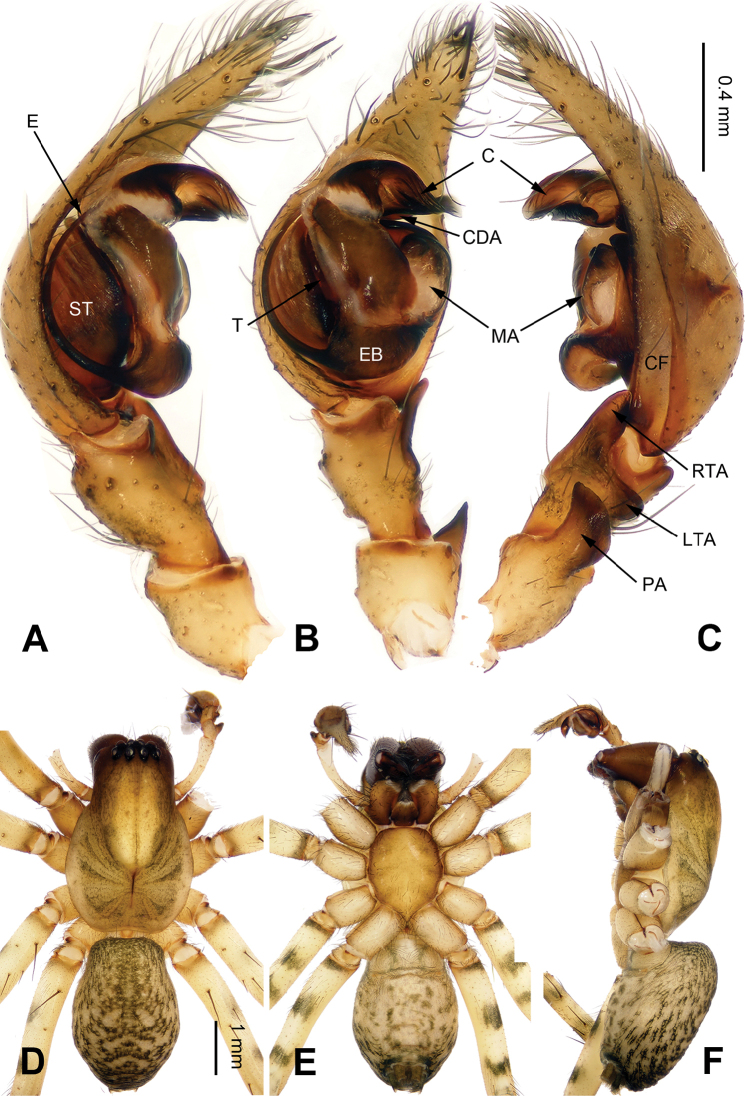
Male palp and habitus of *Sinocoelotes pseudoyunnanensis*. **A** Prolateral **B** Ventral **C** Retrolateral. **D** Habitus, dorsal **E** Habitus, ventral **F** Habitus, lateral. Scale bars: equal for **A, B** and **C**; equal for **D, E** and **F**.

The male has uniquely shaped palps, and can be easily distinguished from all other *Sinocoelotes* gen. n. by the shape of conductor (wave-shaped, broad, and with round-blunt tip in *Sinocoelotes pseudoyunnanensis*, but slenderer and with pointed tip in other species), the longer LTA (about 1/3 length of RTA in *Sinocoelotes pseudoyunnanensis*, less than 1/6 length of RTA in other species), the broader patellar apophysis (the terminal part wider than basal part, and the apex subequal to the width of tibia, the terminal part wider than basal part, but the apex about 1/2 width of tibia in *Sinocoelotes hehuaensis*, the terminal part subequal to, or even slenderer than basal part in other species) (cf. Figs 17A–C and 7A–C, 10A–C, 20A–C).

##### Description.

Described by Wang et al. (2009).

##### Comments.

The species shares a combination of somatic morphology characters with *Sinocoelotes hehuaensis* sp. n. and therefore was assigned to *Sinocoelotes* gen. n. The molecular analysis supports this transfer.

##### Distribution.

China (Yunnan) (Fig. 21).

#### Sinocoelotes thailandensis

Taxon classificationAnimaliaAraneaeAgelenidae

(Dankittipakul & Wang, 2003) comb. n.

Figs 18 , 19 , 21

Coelotes thailandensis Dankittipakul and Wang 2003: 735, figs 24–25 (♂ holotype from Thailand, in MHNG, not examined); Dankittipakul et al. 2005: 7, figs 9–10 (♂♀); Wang et al. 2009: 26, f. 128–142 (♂♀).

##### Material examined.

1♂2♀: Thailand: Chiangmai Province: Mae Cham District, Jeep tract, N18°31'41", E98°29'58", 1649 m, 14.X.2014, H. Zhao, Y. Li and Z. Chen.

##### Diagnosis.

**Figure 18.**
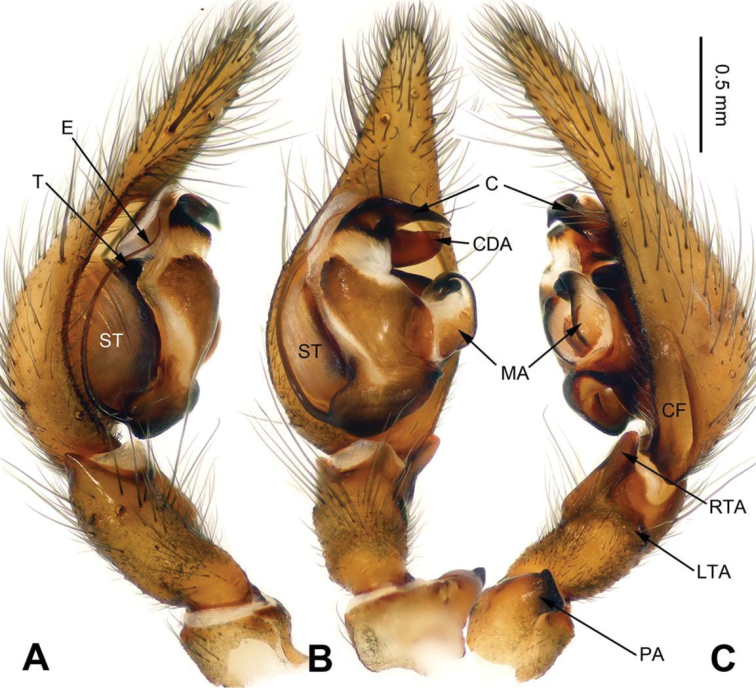
Male palp of *Sinocoelotes thailandensis*. **A** Prolateral **B** Ventral **C** Retrolateral. Scale bar: equal for **A, B** and **C**.

**Figure 19.**
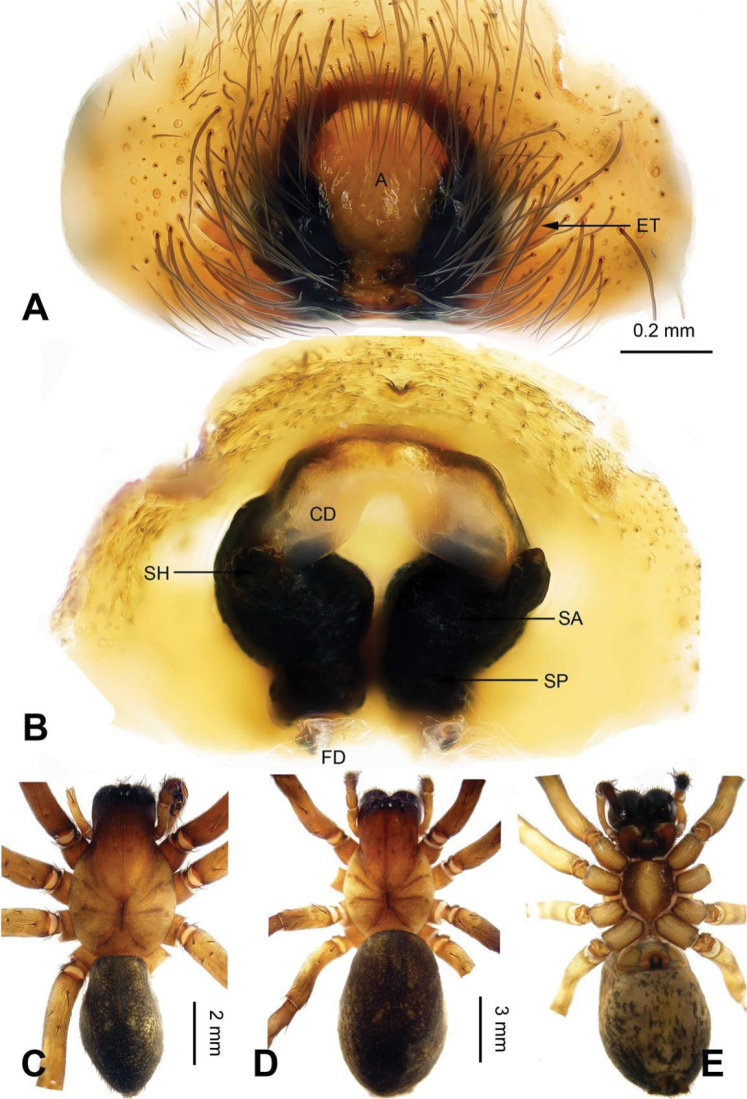
Epigyne and habitus of *Sinocoelotes thailandensis*. **A** Epigyne, ventral **B** Vulva, dorsal **C** Male habitus, dorsal **D** Female habitus, dorsal **E** Female habitus, ventral. Scale bars: equal for **A** and **B**; equal for **C, D** and **E**.

The species is similar to *Sinocoelotes hehuaensis* sp. n., but male can be easily distinguished by a shorter and broader conductor (about 1/3 length of the conductor in *Sinocoelotes hehuaensis* sp. n.), the broad and wedge-shaped dorsal conductor apophysis (cf. Figs 18A–C and 7A–C). The female can be distinguished from that of *Sinocoelotes hehuaensis* sp. n. by the broad (almost round) atrium, the broader and shorter copulatory ducts, the shorter spermathecal heads (about 1/3 length of the spermathecal heads *Sinocoelotes hehuaensis* sp. n.) (cf. Figs 19A–B and 8A–B).

##### Description.

Described by Wang et al. (2009).

##### Comments.

The species shares a combination of somatic morphology characters with *Sinocoelotes hehuaensis* sp. n., and therefore was assigned to *Sinocoelotes* gen. n. The molecular analysis supports this transfer.

##### Distribution.

China (Yunnan) (Fig. 21).

#### Sinocoelotes yanyuanensis

Taxon classificationAnimaliaAraneaeAgelenidae

Zhao & Li sp. n.

http://zoobank.org/B8448C52-A2F2-4E93-8F74-7D6A86A41DD2

Figs 20 , 21

##### Type material.

**Holotype** ♂: China: Sichuan Province: Yanyuan County, foot of Bailing Mountain, in the apple garden, N27°24'03", E101°31'47", 2620 m, 15.XI.2013, Y. Li and J. Liu. **Paratype**: 1 ♂, same data as holotype.

##### Etymology.

The specific name refers to the type locality; adjective.

##### Diagnosis.

**Figure 20.**
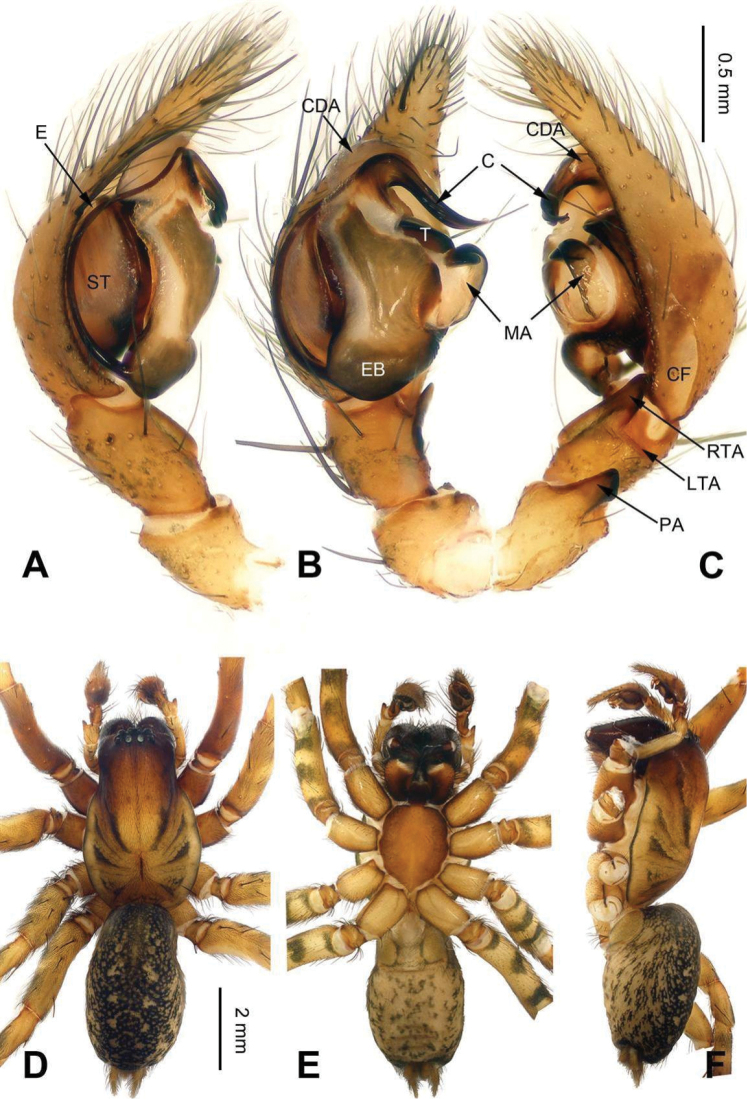
Male palp and habitus of *Sinocoelotes yanyuanensis* sp. n., holotype. **A** Prolateral **B** Ventral **C** Retrolateral. **D** Habitus, dorsal **E** Habitus, ventral **F** Habitus, lateral. Scale bars: equal for **A, B** and **C**; equal for **D, E** and **F**.

The male of the new species has uniquely shaped palps, and can be easily recognized from all other *Sinocoelotes* gen. n. by the clavate patellar apophysis, and the basal part broader than terminal part (bended and 1.5 times as width as *Sinocoelotes yanyuanensis* sp. n. in *Sinocoelotes thailandensis*, basal part equal to or even slenderer than terminal part in other species), the broader and bended conductor in ventral view (wave-shaped in *Sinocoelotes pseudoyunnanensis*, straight in other species), short cymbial tip about 1/4 length of cymbium (about 1/3 length of cymbium in other species), the smaller visible part of dorsal conductor apophysis (quite distinct in other species) (cf. Figs 20A–C and 7A–C, 10A–C, 17A–C).

##### Description.

**Male (holotype)**: Total length 8.55. Carapace 4.35 long, 2.91 wide. Abdomen 4.20 long, 2.50 wide. Eye sizes and interdistances: AME 0.13, ALE 0.19, PME 0.16, PLE 0.13; AME-AME 0.08, AME-ALE 0.04, PME-PME 0.11, PME-PLE 0.19. Leg measurements: I 12.43 (3.40, 4.00, 3.08, 1.95); II 10.80 (2.95, 3.45, 2.65, 1.75); III 10.04 (2.80, 3.16, 2.68, 1.40); IV 13.35 (3.60, 4.25, 3.80, 1.70). Chelicerae with three promarginal and four retromarginal teeth. Palp: patellar apophysis long, subequal to the length of patella, basal part broader than terminal part; LTA short, about1/6 length of RTA; cymbial furrow short, about 1/4 length of cymbium; conductor broader and long, about 1/3 length of cymbium; dorsal conductor apophysis broad, covered mostly by the tegulum and the base of conductor; embolus beginning at 7 o’clock position (Fig. 20A–C).

**Female.** Unknown.

##### Distribution.

**Figure 21.**
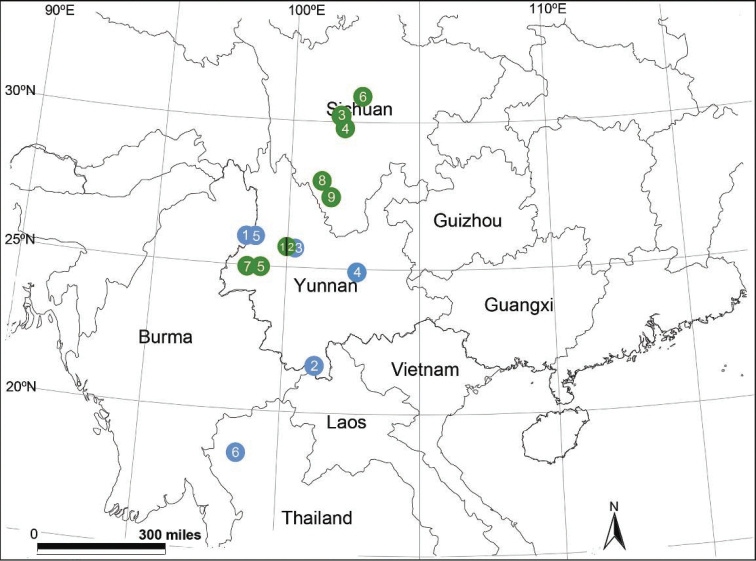
Localities of new (green) and earlier described (blue) species of *Sinocoelotes* gen. n. from China and Thailand. Green: **1** *Sinocoelotes cangshanensis* sp. n. **2** *Sinocoelotes hehuaensis* sp. n. **3** *Sinocoelotes kangdingensis* sp. n. **4** *Sinocoelotes ludingensis* sp. n. **5** *Sinocoelotes luoshuiensis* sp. n. **6** *Sinocoelotes mahuanggouensis* sp. n. **7** *Sinocoelotes mangbangensis* sp. n. **8** *Sinocoelotes muliensis* sp. n. **9** *Sinocoelotes yanyuanensis* sp. n. Blue: **1** *Sinocoelotes acicularis* **2** *Sinocoelotes forficatus* **3** *Sinocoelotes guangxian* **4** *Sinocoelotes pseudoterrestris* **5** *Sinocoelotes pseudoyunnanensis* **6** *Sinocoelotes thailandensis*.

Known only from the type locality (Fig. 21).

## Supplementary Material

XML Treatment for Sinocoelotes

XML Treatment for Sinocoelotes acicularis

XML Treatment for Sinocoelotes cangshanensis

XML Treatment for Sinocoelotes forficatus

XML Treatment for Sinocoelotes guangxian

XML Treatment for Sinocoelotes hehuaensis

XML Treatment for Sinocoelotes kangdingensis

XML Treatment for Sinocoelotes ludingensis

XML Treatment for Sinocoelotes luoshuiensis

XML Treatment for Sinocoelotes mahuanggouensis

XML Treatment for Sinocoelotes mangbangensis

XML Treatment for Sinocoelotes muliensis

XML Treatment for Sinocoelotes pseudoterrestris

XML Treatment for Sinocoelotes pseudoyunnanensis

XML Treatment for Sinocoelotes thailandensis

XML Treatment for Sinocoelotes yanyuanensis
